# 5-Aminolevulinic acid as an emerging radiosensitizer for radiodynamic therapy in solid tumors: a systematic review of available data and clinical potential

**DOI:** 10.1007/s00066-025-02420-0

**Published:** 2025-06-17

**Authors:** Niklas B. Pepper, Fabian M. Troschel, Walter Stummer, Hans T. Eich

**Affiliations:** 1 Department of Radiation Oncology, University Hospital Muenster, Albert-Schweitzer-Campus 1a, 48149 Münster, Germany; 2 Department of Neurosurgery, University Hospital Muenster, Münster, Germany

**Keywords:** 5‑ALA, Radiotherapy, Glioma, Skin cancer, Photon irradiation

## Abstract

**Background:**

5‑Aminolevulinic acid (5-ALA) is a keto-carbon amino acid frequently used in glioma surgery for fluorescence-guided resection. Additionally, cytotoxic properties of 5‑ALA can be induced via stimulation with laser light in photodynamic therapy (PDT). Preclinical in vitro and in vivo trials have also demonstrated this effect to be inducible by photon irradiation as used in radiation treatment. This makes 5‑ALA a potential sensitizer for radiation therapy whose capabilities and limitations have not yet been fully evaluated. In this article, we present results from a systematic literature review regarding the evidence of 5‑ALA’s radiosensitizing properties and the context of its use. We discuss these findings in terms of the underlying mechanisms, their limitations, and the questions to be addressed in future clinical trials.

**Methods:**

A systematic review in the PubMed database was performed via a specifically designed search term, including all search results that featured the combination of 5‑ALA with ionizing radiation. The last date of search was November 13, 2024. Risk of bias among study data was assessed individually according to the study setup after full-text analysis. The results were synthesized based on the underlying tumor entity.

**Results:**

A total of 31 articles were included that examined the combination of 5‑ALA with radiotherapy (RT) in glioma (*n* = 12), melanoma (*n* = 6), breast (*n* = 3), lung (*n* = 2), prostate (*n* = 4), and colorectal (*n* = 1) cancer as well as in sarcoma (*n* = 2) and primary CNS lymphoma (*n* = 1). The radiosensitizing effect of 5‑ALA varies among these entities, with glioma and melanoma presenting the strongest body of evidence.

**Conclusion:**

These results imply a basis for 5‑ALA as a possible radiosensitizer for RT, but several questions remain unanswered, as limitations arise from the fact that data are predominantly based on in vitro or rodent in vivo trials, with only two ongoing clinical trials and one case report involving human patients. Moreover, trial setups varied in terms of ALA dose and application timing.

**Supplementary Information:**

The online version of this article (10.1007/s00066-025-02420-0) contains supplementary material, which is available to authorized users.

## Introduction

5‑Amniolevulinic acid (5-ALA) is an amino acid of the keto-carbon group. As a substrate in cellular heme synthesis, it is used to create porphobilinogen (PBG) in the mitochondrial matrix, which is then converted to protoporphyrin IX (PpIX) and, sequentially, to heme in normal cells. The value of 5‑ALA in oncological treatment derives from an accumulation of PpIX in tumor or tumor-like tissue [1], based on increased uptake and altered heme synthesis pathways. Stimulation of the photoactive PpIX with light of a certain wavelength can trigger a fluorescent effect which is used in glioma surgery to discriminate between tumorous and healthy tissue. In this context, fluorescence-guided surgery has been shown to improve resection outcomes and enhance survival [2]. Additionally, stimulation with laser light can trigger a cytotoxic effect which is harnessed in photodynamic therapy (PDT) and has been established in a variety of cancers [3–7]. The need for exposure to laser light has been a limitation to PDT, since this is only feasible in intraoperative settings and for cutaneous or endoscopically accessible tumors such as tumors of the lung, esophagus, stomach, and bladder as well as gynecological or oral lesions [6]. To overcome the limitation of direct exposure, the possibility of treating PpIX-enriched cells with tissue-penetrating photons (i.e., X‑rays or MV photon irradiation) has been explored and was demonstrated successfully in vitro and in vivo [8–10]. In analogy to PDT, the concept has been referred to as radiodynamic therapy (RDT): the combination of 5‑ALA and ionizing radiation leads to an increased cytotoxic effect based on a series of cellular mechanisms. After administration of the drug, it enters the cell through specific amino acid transport channels (namely ABCB6, PepT1/2, and transporters of the SLC6 family), the upregulation of which in malignant cells contributes to a tumor-selective effect [11–13]. Inside the cell, 5‑ALA enters the heme biosynthesis pathway inside the mitochondria and is metabolized to protoporphyrin IX (PpIX), which accumulates in malignant cells due to their altered mitochondrial activity and increased heme biosynthetic activity, leading to further selective activity [14].

When exposed to ionizing radiation (IR), a cascade of processes unfolds: reactive oxygen species (ROS) such as hydrogen peroxide (H_2_O_2_), hydroxyl radicals (•OH), and superoxide anions (O_2_•-) are generated via radiolysis of water molecules. Energy absorption by the accumulated PpIX leads to its excitation into a singlet state which, in turn, produces additional ROS via electron transfer reactions when returning to the ground state [15]. Interestingly, the amount of and ratios between different species seem to be dependent on O_2_ saturation, as hydroxyl radicals have been shown to increase, regardless of the dissolved oxygen, while superoxide increases proportionally [16]. Sequentially, ROS lead to various types of cellular damage in biomolecules such as lipids, proteins, and DNA itself as well as to disruption of mitochondrial function [17]. This prompts a further increase in ROS generation, inducing a cycle of oxidative stress [18, 19]. This is irreparable to the cell, as it leads to DNA double-strand breaks (DSBs) and consecutive cell cycle arrest in the S and G2/M phase as well as membrane disruptions, thus inducing apoptosis or necrosis based on the level of stress [17]. Additionally, apoptosis can be induced by an increase in intercellular cytochrome c caused by disruption of the mitochondrial membrane potential leading to increased permeabilization.

**Fig. 1:**
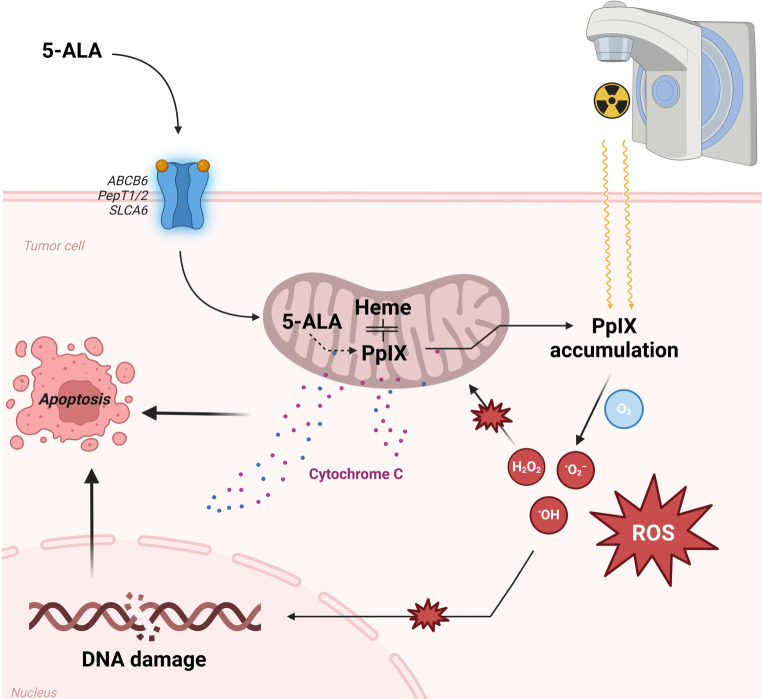
Mechanism of the radiosensitizing effect of 5‑aminolevulinic acid (5-ALA) in tumor cells. *PpIX* protoporphyrin IX, *ROS* reactive oxygen species. Created with biorender.com

Thus, these synergistic mechanisms enhance the cytotoxic capabilities of ionizing radiation in the presence of 5‑ALA. Furthermore, arrest in the G2/M phase may lead to increased sensitivity of cells to ionizing radiation (IR) by sequential RT fractions, as this state is highly vulnerable to IR-mediated cell damage. The mechanism is illustrated in Fig. 1.

While preclinical data have delivered promising results, the value of 5‑ALA has not yet been fully explored; it is under investigation in contemporary clinical and preclinical trials. In this article, we focus on the use of 5‑ALA as a radiosensitizer for photon irradiation. Our objective is to systematically review and discuss the available data for ALA-RDT in different malignancies as well as the uncertainties and limitations of this emerging concept.

## Methods

This article was prepared following the Preferred Reporting Items for Systemic Review and Meta-Analysis (PRISMA) guideline [20]. The corresponding PRISMA checklist can be found in the Electronic Supplementary Material; a review protocol was not prepared.

We performed a systematic review in the PubMed database with the search term “5-ALA [All] AND (photon irradiation [All] OR radiotherapy [All] OR radiodynamic therapy [All]).” The last date of consultation was November 13, 2024. All abstracts identified by this search term were included in the analysis, screened for accessibility, and reviewed. Articles were then excluded if they did not cover treatment involving RDT (but rather PDT, sonodynamic therapy [SDT], or fluorescence-guided resection) or if they were limited to background information (comments on other articles or letters regarding trial designs). When in doubt, studies underwent full-text analysis. Two different radiation oncologists performed the full-text analysis as a means of verification of the inclusion process to validate the article selection; no automation tools were used, and no data were directly obtained from study investigators. The search strategy as well as the inclusion and exclusion criteria are summarized in Table 1. After completing the selection process, a full-text analysis of all included articles was performed, and data regarding trial setup, ALA application, specifics of radiotherapy, and results of the investigation were collected by one reviewer. If additional variables were of interest, they were also selected for the discussion (e.g., identified roadblocks and confounders). Risk of bias and reasons for heterogeneity among study data were assessed individually according to the study setup and discussed in light of all available data after full-text analysis. The trials were grouped based on the examined tumor entity for discussion.

**Table 1:** Search strategy and inclusion and exclusion criteria of the systematic literature review. *5-ALA* 5-aminolevulinic acid, *RDT* radiodynamic therapy, *PDT* photodynamic therapy, *SDT* sonodynamic therapy

*Search strategy*
Database: PubMed
Search algorithm: 5‑ALA [All] AND (photon irradiation [All] OR radiotherapy [All] OR radiodynamic therapy [All])
*Inclusion criteria*
All abstracts identified by the search strategy were included
*Exclusion criteria*
Articles featuring fluorescence-guided resection rather than RDT
Articles featuring PDT or SDT rather than RDT
Articles without distinctive data (e.g., comment or review)
Articles not available in English

## Results

**Fig. 2:**
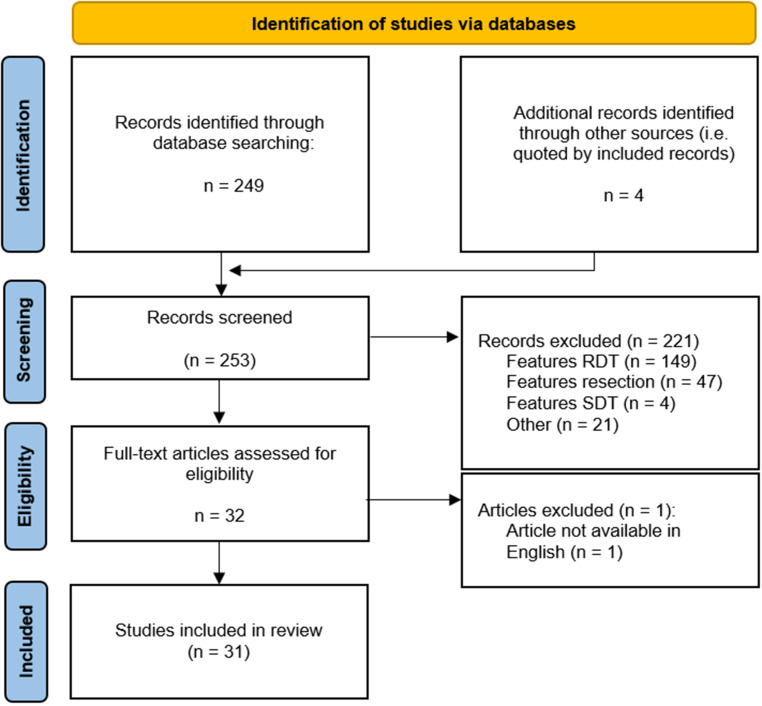
Study screening and inclusion during the literature review process as recommended by the PRISMA guideline

Our search strategy generated 249 results, of which 28 abstracts were included for full-text study, and 221 were excluded from further analysis. Reasons for exclusion were articles featuring ALA-PDT rather than RDT (*n* = 149), articles featuring fluorescence-guided resection with 5‑ALA rather than RDT (*n* = 47), articles featuring ALA-SDT rather than RDT (*n* = 4), or other reasons (e.g., only comments on other articles, *n* = 21). During the screening process, 4 more articles were included which were not identified by the initial search strategy but were cited by articles previously included. The remaining 32 articles were evaluated in full. During full-text analysis, one additional article which was not accessible in English was excluded. Figure 2 illustrates the selection process as a flowchart based on the PRISMA recommendations. Overall, 8 included articles reported in vitro data, 11 in vivo data, 8 in vitro and in vivo data, 1 article was a case report retrospectively analyzing a pediatric case, and 2 articles reported the protocols of ongoing prospective clinical trials. Both prospective trials are phase I/II trials. The included articles were synthesized for discussion by the tumor entity treated in the respective trials: 12 articles were attributed to the field of glioma (with 7 covering glioblastoma), 6 address melanoma, 3 breast cancer, 2 lung cancer (evenly distributed between small cell and non-small cell lung cancer 1:1), 4 prostate cancer, 1 colorectal cancer, 2 sarcoma, and 1 cerebral lymphoma. The ongoing prospective trials explore the use of 5‑ALA in combination with re-irradiation for recurrent glioblastoma (one trial) and with brachytherapy for prostate cancer (also one trial). Scrutinizing the individual reported setups of the included articles, the applied doses of 5‑ALA ranged from 50 to 300 mg/kg and the prescribed radiation dose varied between single- and multi-fraction RT, with daily doses between 2 and 20 Gy. Regarding endpoints, 7 studies reported increased radiosensitivity in their respective setups, an increase in generation of reactive oxygen species was reported by 8, a delay in tumor growth by 13, an increase in specimen survival by 3, and a benefit in terms of RT tolerability was reported by 2 studies (with some articles reporting multiple endpoints). In contrast, 3 studies found no evidence of enhanced RT effectiveness after treatment with 5‑ALA and another 3 found a positive effect only when combining 5‑ALA with an additional substance, but not as a single agent. Of note, 16 of the excluded manuscripts featuring PDT rather than RDT in indications for which RT is a key component in the treatment strategy were also summarized in “Future perspective entities” in the discussion. Table 2 summarizes the included articles which are discussed in the following based on the treated tumor entity. Heterogeneity among study results is also discussed in the respective segments.

**Table 2:** Results of the systematic literature review

First author	Year	Tumor entity	Setup	Cell line or type	ALA dose	RT dose	Observed effect of 5‑ALA + RT	Reference
Yamamoto J	2012	GBM	In vitro	9L/C6	1 mM, 4 h prior to RT	Up to 10 Gy in 5 fx	Increased radiosensitivity and ROS production	[22]
Yamamoto J	2015	Glioma	In vivo	9L	100 mg/kg i.v. injection, 3h prior to RT	10 Gy in 5 fx	Significant delay in tumor growth	[10]
Kitagawa T	2015	Glioma	In vitro	9L/U251	1 mM, 4 h prior to RT	10 Gy in 1 fx	Significant increase in ROS production	[9]
Park Y	2016	Glioma	In vitro and in vivo	MES-GSCs/NP-GSCs/MES-GSCs	240 mg/kg i.p. injection	30 Gy in 10 fx	Significant increase in median survival	[25]
Ueta K	2016	Glioma	In vitro	9L/U251	Up to 1 mM, 24 h prior to RT	Up to 10 Gy in 1 fx	Increased cell death and increased production of delayed mitochondrial ROS	[23]
Takahashi J	2021	GBM	In vivo	U251/U87	Up to 240 mg/kg orally, 4 h prior to RT	60 Gy in 30 fx	Slowed tumor progression and promoted regression with no significant increase in toxicity	[70]
Chiang CS	2021	GBM	In vitro and in vivo	GBM8401/GL261	20 mg/kg i.c. injection, 24 h prior to RT	2 Gy in 1 fx	Significant reduction of cell viability in combination with a spherical radioenhancer (GD)	[73]
Dupin C	2022	GBM	In vivo	Human GBM cells	100 mg/kg i.p. injection, 4 h prior to RT	Up to 15 Gy in 5 fx	No enhancement of radiation efficiency	[26]
Fukumura M	2023	GBM	In vitro and in vivo	HGG13/HGG30/TS	80 mg/kg orally, 4–26.5 h prior to RT	Up to 21.9 Gy Eq in 1 fx	Improved survival and upregulation of amino acid transporters	[24]
Gordon JA	2023	Glioma	Case report	Human HGG cells	N/A	60 Gy BTX	Effective radiotherapy dose localization with sparing of organs at risk	[74]
Mandl GA	2023	GBM	In vitro	U251	1 mM, 24 h prior to RT	Up to 20 Gy in 1 fx	Increased radiosensitizing effect in combination with Pr3+ nanoparticles	[75]
Pepper NB	2024	GBM	Clinical trial	Human GBM	20 mg/kg orally, 6–8h prior to RT	Up to 39.6 Gy in 22 fx	Results pending	[28]
Takahashi J	2013	Melanoma	In vitro and in vivo	B16-BL6	50 mg/kg i.t. injection, 24 h prior to RT	30 Gy in 10 fx	Significant delay in tumor growth	[29]
Takahashi J	2016	Melanoma	In vivo	B16-BL6	50 mg/kg i.t. injection, 24 h prior to RT	30 Gy in 10 fx	Significant delay in tumor growth and dysregulation of genes related to cell cycle arrest	[30]
Mohammadi Z	2017	Melanoma	In vitro	MeL-Rm	20 mM, 24 h prior to RT	Up to 16 Gy in 1 fx	No enhancement of radiation efficiency in combination with gold nanoparticles	[31]
Takahashi J	2018	Melanoma	In vivo	B16-BL6	50 mg/kg i.v. injection, 4–5 h prior to RT	Up to 30 Gy in 10 fx	Slowed tumor progression and promoted regression	[8]
Takahashi J	2019	Melanoma	In vivo	B16-Luc	200 mg/kg i.p. injection, 4 h prior to RT	14 Gy in 7 fx	Significant delay in tumor growth	[17]
Hasegawa T	2020	Melanoma	In vitro	B16	5 µM PpIX	Up to 10 Gy in 1 fx	Increase in ROS production, single-strand and double-strand breaks	[16]
Kaneko T	2018	Breast	In vitro	EMT6	1 mM, 5 h prior to RT	Up to 1.5 Gy Eq in 1 fx	Increase in mitochondrial ROS production and double-strand breaks	[37]
Jiang F	2022	Breast	In vitro	4T1	N/A	5 Gy in 1 fx	Significantly improved tumor suppression and survival in combination with RDT with the CsI(Na)@MgO nanoparticles but no enhancement of radiation efficiency of ALA alone	[35]
Viswanath D	2023	Breast	In vivo	4T1	1 mM, 5 h prior to RT	8 Gy in 1 fx	Significantly improved survival in combination with calcium tungstate nanoparticles but no enhancement of radiation efficiency of ALA alone	[36]
Yang DM	2022	SCLC	In vivo	KP1	100 mg/kg i.v. injection, 4 h prior to RT	4 Gy in 1 fx	Significant delay in tumor growth	[38]
Han J	2022	NSCLC	In vivo	H1299	50 mg/kg i.v. injection, 3 h prior to RT	5 Gy in 1 fx	Significant delay in tumor growth, further increase in combination with doxorubicin-coated nanoparticles	[39]
Miyake M	2019	Prostate	In vitro and in vivo	MyC-CaP	Up to 300 mg/kg orally	12 Gy in 3 fx	Significant delay in tumor growth and radioprotective profile in normal rectal and urinary bladders	[32]
Miyake M	2020	Prostate	Clinical trial	Human PC	200 mg per day orally for 6 months	160 Gy BTX	Results pending	[33]
Panetta JV	2020	Prostate	In vivo	PC‑3	100 mg/kg iv injection, 4 h prior to RT	4 Gy in 1 fx	Significant delay in tumor growth in combination with carbamide peroxide but not with ALA alone	[34]
Owari T	2022	Prostate	In vitro and in vivo	PC-3/DU-145/MyC-CaP	30 mg/kg orally, 3 h prior to RT	20 Gy in 10 fx	Significant delay in tumor growth, increased mitochondrial ROS production, induced mitochondrial dysfunction, and decreased radiation resistance under hypoxic conditions	[71]
Yamada K	2019	Colorectal	In vitro and in vivo	HT29	50 mg i.p. injection, 4 h prior to RT	Up to 15 Gy in 5 fx	Significant delay in tumor growth	[48]
Schaffer M	2002	Sarcoma	In vivo	Lewis sarcoma	200 mg/kg injection, 24 h prior to RT	3 Gy in 1 fx	No enhancement of radiation efficiency	[45]
Tung FI	2021	Sarcoma	In vitro	MG63	200 μg/ml, 24 h prior to RT	2gy in 1 fx	Increased ROS production in combination with chitosan-coated nanoparticles but no enhancement of radiation efficiency of ALA alone	[46]
Suzuki K	2023	PCNSL	In vitro	Raji/HKBML/TK	0.3 μM, 4 h prior to RT	Up to 20 Gy in 10 fx	Increased delayed ROS production for all cell lines under normoxic conditions and for TK cells under hypoxic conditions	[15]

*ALA* aminolevulinic acid, *BTX* brachytherapy, *fx* fractions, *GBM* glioblastoma, *i.p.* intraperitoneal, *i.v.* intravenous, *ROS* reactive oxygen species, *RT* radiotherapy, *@* coated with

## Discussion

### Glioma

The largest body of evidence for the radiosensitizing properties of 5‑ALA can be found in the treatment of glioma. Since 5‑ALA is commonly used for fluorescence-guided resection in high-grade glioma and PDT has also shown promising results, it is not surprising that the use of ALA-RDT has been explored in this field by several working groups [1]. After Kostron et al. delivered first results regarding the radiosensitizing properties of hematoporphyrin derivatives in 1986 [21], Yamamoto et al.’s in vitro analysis of tumor growth inhibition and ROS production in rat glioma cell lines (9L gliosarcoma and C6 glioma) exposed to different doses of ionizing radiation after treatment with 5‑ALA revealed the benefit of combined treatment [22]. This effect has been validated by follow-up trials [9, 23] and translated into in vivo settings, revealing the benefit of 5‑ALA with fractioned radiotherapy (e.g., using a standard schedule of daily RT over 6 weeks as implemented in glioblastoma treatment) in murine models [8, 10, 24, 25]. A different working group could not replicate the effect of radiosensitization in an orthotopic model of glioblastoma using cranially implanted tumor spheroids and an in vivo mouse model [26]: in their detailed analysis, Dupin et al. also pointed out limitations of prior studies which did not combine the same host/tumor/graft site and had variations in RT schedules. Recent publications have attributed the absence of a positive effect of RDT to the wide variation in the measured bioluminescence of the brain tumors in the RDT group [15]. Since this might also represent a limitation in clinical settings, further research is needed regarding possible differences in ALA uptake at different tumor sites and in different tumor entities.

Two trials involved human patients: in a phase I study, Schaffer et al. treated 12 patients with solid tumors at different sites (including 3 high-grade gliomas) with radiotherapy and upfront injection of Photofrin II (initially provided by Axcan Pharma Inc, Mont-Saint-Hilaire, Canada; now distributed by Concordia Laboratories Inc. Pinnacle Biologics, Bannockburn, IL, USA), a commercially available hematoporphyrin derivate. Of the 3 glioma patients, 2 showed stable disease for > 12 months and one developed early relapse [27]. Of note, the other patients with different solid tumors (including bladder cancer, sarcoma, and oropharyngeal tumors) also responded well to treatment and showed stable disease or partial remission with few side effects. An ongoing phase I/II dose-escalation trial is using multiple applications (up to eight times 20 mg/kg) of 5‑ALA during the course of re-irradiation for recurrent GBM [28]. Using a dose-escalation approach, this trial will deliver important data not only for glioma patients but rather for all entities with possible implementation of PpIX-mediated radiosensitization, since no trials have yet implemented a maximum tolerated dose with repeated ALA applications. However, these might be necessary because prior in vitro trials have shown that the concentration of PpIX substantially drops 24 h after application [26].

The existing preclinical evidence and setup of a first prospective phase 1 trial make the field of glioma the entity most likely to see an implementation of ALA-RDT in the future. However, several questions remain unanswered, as discussed in the “Uncertainties and possible limitations of ALA-RDT” section.

### Melanoma

5‑ALA is commonly used as a mediator of PDT in the treatment of skin diseases [5]. Several preclinical in vitro and in vivo trials have also demonstrated the effectiveness of RDT when treating melanoma cell lines: Takashi et al. did not only demonstrate a reduction in tumor growth in vivo [17, 29, 30] but also translated the setup to treatment with a linear accelerator (LINAC) as compared to a laboratory X‑ray irradiator [8], showing that treatment of patients with LINACs as used in radiation oncology clinical routine is feasible in an RDT setting with 5‑ALA as a photosensitizer. Interestingly, not all melanoma cell lines seem to respond to ALA-RDT: Mohammadi et al. found a positive effect for PDT, but no increase of RT effectiveness in the treatment of MeL-RM cells (as opposed to Takahashi et al. using B16-BL6 melanoma cells) when using ALA-plated gold nanoparticles prior to irradiation [31]. Hasegawa et al. validated that the cell-killing properties of X‑ray irradiation can be increased in the presence of PpIX, as they demonstrated an increase in DNA single- and double-strand breaks [16]. Since this effect is mainly mediated by ROS in the form of peroxide (•OH) and superoxide (O_2_•-), it is important to note that the increase in cellular (O_2_•-) in this trial was proportional to the dissolved oxygen, while (•OH) was independent of it. Thus, ensuring a high level of O_2_ saturation might increase the effectiveness of ALA-RDT, which should be considered in future clinical trials.

### Prostate cancer

Radiotherapy is a common treatment option for prostate cancer in different scenarios. Especially in early-stage disease, external-beam radiotherapy (EBRT), brachytherapy (BTX), or a combination of both offer excellent degrees of long-term disease-free survival. With modern RT techniques like image-guided RT (IGRT), intensity-modulated RT (IMRT), and daily adaptation of target volumes (ART), high levels of precision have become standard. However, due to a need for high doses in a target volume adjacent to vulnerable organs at risk, the reduction of treatment toxicity remains a focus of clinical research. In this regard, a study by Miyake et al. not only demonstrated a radiosensitizing effect in human and murine prostate cancer cells but could also show a radioprotective effect for rectal and urinary tract mucosa, potentially representing a double benefit of ALA in the treatment of prostate cancer [32]. Consequentially, a currently ongoing clinical trial with human prostate cancer patients was designed, wherein patients are treated with a standard prescription dose of 160 Gy of iodine-125 brachytherapy (BTX) while harnessing the effects of RDT as patients are administered oral 5‑ALA in combination with sodium ferrous citrate as capsules twice a day for 6 months during treatment [33]. Future trials might also incorporate RDT during treatment with EBRT. Panetta et al. confirmed that the antitumor effect of PpIX-enhanced irradiation is also prevalent in a mouse model (using mice implanted with human PC‑3 prostate cancer cells) when using a combination of 5‑ALA and carbamide peroxide before treatment with a 15-MV LINAC, thereby delivering important data for the transition of laboratory setups to clinical application [34]. While a significant effect on survival and toxicity will be hard to determine in prostate cancer as modern therapeutic approaches for early-stage disease already offer high levels of freedom from treatment failure with few overall side effects, the combination of ALA with BTX offers a highly interesting perspective, circumventing the difficulties of drug and RT application timing. Combined with the dual benefit of increased antitumor efficacy and protective effects on mucosa, the limited existing data also point towards a fruitful combination to be explored, and results of the prospective trial should be highly anticipated in that regard.

### Breast cancer

For women receiving breast-conserving surgery, postoperative RT is the standard of care, making breast cancer (as well as the previously outlined prostate cancer) one of the most frequent indications for radiation oncologists. Evidence regarding a benefit of 5‑ALA is limited, consisting of two studies of nanoparticle-based delivery of 5‑ALA before irradiation in 4T1 cells, which present improved tumor suppression when combining the respective models with ionizing radiation in vitro [35] and in vivo [36]. However, no enhancement of radiation efficiency was seen for 5‑ALA alone. Finally, one in vitro study confirmed a significant radiosensitizing effect for EMT6 tumor cells when combining 5‑ALA with carbon ion beam irradiation [37]. The heterogeneity in these reports is most likely attributed to the differences in trial setups: Kaneko et al. [37] were able to demonstrate a radiosensitizing effect of 5‑ALA alone when using carbon ion irradiation, which has a higher linear energy transfer than standard photon irradiation, while no effect of 5‑ALA alone was found by Jiang et al. [35] and Viswanath et al. [36] using a radioresistant breast cancer cell line. Nevertheless, both the latter studies found a significant effect when combining ALA-RDT with different, specifically designed nanoparticles. Nevertheless, ALA played a role as a mediator of cytotoxicity, as controls using RT and the respective nanoparticles without 5‑ALA did not show a benefit over RT alone. Due to the limited amount of data, more evidence is needed in this entity.

### Lung cancer

Radiotherapy in the form of (neo)adjuvant or definitive chemoradiotherapy (CRT) as well as monomodal stereotactic RT is commonly used in curative treatment of lung cancer patients. Especially cases with mediastinal involvement could potentially benefit from a selective increase in RT effectiveness in tumor cells, due to the close proximity to organs at risk (e.g., heart and esophagus). Two preclinical trials have demonstrated the use of 5‑ALA as a radiosensitizer for lung cancer: Yang et al. used an in vivo model with KP1 SCLC cells, showing a significant delay in tumor growth, increased survival, and reduction of fluorodeoxyglucose (FDG) uptake after injection of 100 mg/kg 5‑ALA 4 h prior to single-fraction RT with 4 Gy [38]. Their approach to using 15-MV irradiation underscores the possibility of using MV RT as commonly applied in clinical practice with human patients to trigger an RDT effect. Han et al. combined 5‑ALA application prior to single-fraction 5 Gy irradiation with doxorubicin-coated nanoparticles in H1299 NSCLC cells in vitro and in vivo [39]. They found a significant increase in double-strand breaks in vitro and increased tumor growth suppression via RDT. This effect was amplified in the group simultaneously treated with doxorubicin-coated nanoparticles, rendering harnessing of the RDT effect in the context of CRT possible as well, especially since no signs of increased toxicity were found. Nevertheless, experiments including multi-dose or even accelerated RT schedules (as often applied in the treatment of lung cancer) have not been conducted.

### Lymphoma

Primary central nervous system lymphoma (PCNSL) is a rare disease with a mostly poor outcome: low-grade entities account for only a very small fraction, while the majority of cases comprise aggressive subtypes (usually diffuse large B‑cell lymphoma). PCNSL is highly radiosensitive, and indolent subtypes may be treated with focal RT alone [40]. For the more common high-grade subtypes, whole-brain irradiation (WBI) is a preferred concept to adequately address the infiltrative nature of the disease. However, WBI has gradually been replaced by complex systemic treatment because of its high potential for neurotoxicity. Low-dose WBI is an emerging concept to avoid these sequelae, and the combination of modern systemic approaches with focal radiotherapy might have potential, but evidence is lacking at the moment [41]. In this context, a radiosensitizing agent could optimize antitumor effectiveness while ensuring minimized toxicity. Additionally, several authors have described strong ALA-induced fluorescence in cerebral lymphoma [42–44]. One study by Suzuki et al. evaluated the potential of combining 5‑ALA and RT in lymphoma cells (Raji, HKBML, and TK) in vitro under hypoxic and normoxic conditions [15]. To do so, they evaluated the production of PpIX and ROS as well as the cytotoxic effect after a single irradiation with 2/8 Gy via a gamma irradiator. They found accumulation of 5‑ALA-generated PpIX, and the survival rate of cells treated with 5‑ALA and RT was significantly decreased when compared to the control group treated with RT only under normoxic and hypoxic conditions. A delayed increase in ROS production 12 h after RT was observed in all cell lines when treated with 5‑ALA (compared to untreated control). Under hypoxic conditions, this effect was only reproducible for one of three cell lines (TK), again emphasizing the need for oxygen saturation to optimally trigger the radiosensitizing effect of 5‑ALA.

### Sarcoma

In 2002, Schaffer et al. compared the radiosensitizing capabilities of Photofrin II (another mediator of PDT increasing ROS via accumulation of PpIX) to 5‑ALA in a rodent model using a Lewis rat sarcoma cell model. While an effect was found after 1 × 3 Gy RT for Photofrin, no effect was seen after pretreatment with 5‑ALA [45]. Moreover, 19 years later, Tung et al. found an increase in ROS when combining RT, ALA, and chitosan-coated gold nanoparticles in osteosarcoma cells in vivo, but, once again, mere application of ALA before RT did not increase the response to RT [46]. Therefore, 5‑ALA might serve as a mediator, but the evidence does not favor using it as a single agent for RDT in sarcoma.

### Colorectal cancer

While colon cancer is typically treated with surgery and chemotherapy, RT is regularly administered in the treatment of rectal cancer for locally advanced disease as part of neoadjuvant treatment (in the form of short-course RT, long-course CRT, or intensified total neoadjuvant treatment). Modern concepts also focus on organ preservation, exploring inducing complete remission via an increase in focal treatment dose [47]. These promising concepts could potentially benefit from tumor-selective enhancement of RT to reduce damage in normal tissue. Yamada et al. were able to demonstrate a reduction in tumor growth in vivo after multiple irradiations in mice implanted with human HT29 colon cancer cells after previous intraperitoneal injection with 5‑ALA [48]. Interestingly, the RDT effect was only prevalent after multi-fraction and not after single-fraction RT. Of note, Kamada et al. also reported reduced tumor growth after multiple RT fractions in mice implanted with human HT29 colon cancer cells when pretreated with 5‑ALA. However, the article was not accessible for full-text evaluation and, therefore, not included in Table 2, since important data like ALA dose and RT specifications remain unknown [49]. While the existing data are very limited at the moment, additional benefit might also derive from the protective properties for normal mucosa, as demonstrated by Miyake et al. in prostate cancer [32].

### Future perspective entities

Of the excluded manuscripts, 16 articles featured entities with potential RT indications. Since the use of PDT might translate into RDT, these entities are summarized in the following.

In meningioma, radiotherapy plays a key role in the treatment of high-grade, unresectable, or recurrent tumors, with a variety of possible fractioned or stereotactic RT application methods and dose schedules. As 5‑ALA has already been explored in the context of fluorescence-guided resection and PDT for meningioma as well [50–52], RDT might also be a relevant treatment option, especially in high-grade or pretreated scenarios of critical location where the RT dose might be limited due to the dose constraints of healthy tissue. Other entities with frequent indications for definitive or adjuvant irradiation that were evaluated for PDT include tumors of the head and neck region [53–56], esophageal cancer [57], gynecological malignancies, and premalignancies such as cervical cancer [58–61], hepatocellular cancer [62], bladder cancer [63], and different types of skin neoplasia [64–68]. Since PDT is more implemented than the emerging concept of RDT, these trials in part also include human patients, showing good tolerability and effectiveness. In skin diseases, a combination of PDT and RT has also been explored, with encouraging results [67]. Therefore, future trials might also incorporate RDT into radio-oncological concepts in these entities.

### Uncertainties and possible limitations of ALA-RDT

The included studies consist mainly of preclinical trials and therefore have a low risk of bias. While the majority of publications could demonstrate a benefit of combining 5‑ALA with ionizing irradiation, some authors did not find a synergistic effect or, in some cases, an additional agent was needed to trigger an increase in radiosensitivity [34–36, 46]. The heterogeneity of these study data might arise from differences in trial setups, leading to limitations and uncertainties regarding combined use in future endeavors.

The first and most fundamental uncertainty is the overall manifestation of a synergistic effect in different tumor entities:

As pointed out by Dupin et al., preclinical models show a variety of implantation sites of tumor cells in vivo. In their approach, using an orthotopic model of glioblastoma cells did not show the radiosensitizing effect described by several other authors under different circumstances [26]. As glioma is the tumor family with the largest body of evidence for 5‑ALA’s radiosensitizing properties, this calls into question the transferability of the existing in vitro and in vivo data to clinical settings. However, the failure to replicate the benefit in this publication was based on detection of tumoral bioluminescence, which may have underestimated the effect due to the range of results in treatment as well as control groups, as previously discussed by Suzuki et al. [15]. Nevertheless, these data question the level of confidence in ALA-RDT promoted by other authors and the transfer to everyday clinical practice, even in tumors with a relatively high body of evidence (such as glioma and melanoma), as the individual effectiveness might be impaired based on varying oxygen levels, genomic alterations, tumor heterogeneity, and individual expression of amino acid transporter proteins and heme biosynthesis enzymes, as illustrated by Ebrahimi et al. [69]. These variabilities are hardly projectable into rodent models, and future trials will need to address these uncertainties.

Additionally, the presented review demonstrates variability in the susceptibility to ALA-RDT in different tumor entities. While the existing data do not allow a conclusive explanation for this heterogeneity, it is most likely to be attributed to differences in tumor biology. Again, tissue- and tumor-specific differences in cell proliferation, intracellular metabolism, oxygen supply, and amino acid channel structures might be the underlying reasons. The articles discussed in this review might depict melanoma and glioma as the most susceptible to ALA-RDT, but this is likely biased by the relatively larger number of publications in these entities, for which 5‑ALA has already been implemented in the context of PDT and/or fluorescence-guided resection. But as the investigation of this novel combination of RT and radiosensitizer progresses into clinical use, positive results might also kindle additional efforts exploring other entities.

Further uncertainty concerns the optimal manner, dose, and timing of ALA application: again, the included studies show high diversity regarding the form of application (oral, intraperitoneal, and intravenous), the respective dose (ranging from 50 to 300 mg/kg), and the latency between drug administration and irradiation (ranging from 3 to 24 h). Oral 5‑ALA is easily administrable and has demonstrated tissue penetrance even in privileged areas like the brain in the context of PDT and fluorescence-guided resection and has shown a positive radiosensitizing effect in multiple in vivo experiments [24, 32, 70, 71]. Hence, oral admission seems to be the favorable practice and is employed in both ongoing clinical trials [28, 33].

The optimal timing and dose of drug administration also remain elusive, as the presented trials do not enable correlation of the effectiveness of ALA-RDT with certain parameters. The time between drug application and irradiation varies between 3 and 24 h prior to RT. Again, different tumors are likely to need different solutions based on tissue penetration. For glioma, data on fluorescence kinetics point towards a peak of mediating PpIX after 7–8 h [72], but no data involving human patients are available for other tumor entities. Dupin et al. highlighted that different entities might be in need of individual RDT solutions, as cell proliferation and growth capacity differ between tumors [26]. An approach with BTX as used in the AMBER trial [33] circumvents this obstacle via continuous application of IR, but BTX is not implemented for all types of cancer previously discussed; therefore, scheduling of drug and RT application needs careful planning in the setup of future trials. The production of ROS after RDT was evaluated to increase over time (for 12 h) and drastically decreases after 24 h [15]. This renders multiple applications necessary to fully harness the sensitizing capabilities. Repeated exposure to oral 5‑ALA seems safe and well tolerated in vivo, with none of the multi-dose setups showing increased side effects other than mild hepatotoxicity [10]. This is in line with experiences with fluorescence-guided resection, rendering 5‑ALA not only easily applicable (via oral intake) but also well tolerated, with mostly few side effects limited to mild transient increases in liver enzymes and cutaneous sensitivity to light. Regarding dose, Takahashi used orally administrated 5‑ALA in a glioblastoma rodent model with a dose of 240 mg/kg [70] and Miyake et al. even used 300 mg/kg in a murine prostate cancer model [32], both showing positive radiosensitization and no increase in side effects, rendering high-dose applications possible. While the ongoing clinical trials already implement repeated drug administration, both of them use a relatively lower dosage (20 mg/kg for glioblastoma [28], a dose also commonly used for fluorescence-guided resection; and a 200 mg fixed dose for prostate cancer [33]). However, limitations remain, since these clinical trials have not reported safety data yet and no toxicity data regarding multiple applications of 5‑ALA exist from larger trials. Additionally, monitoring and reporting of toxicity in the in vivo trials might not have been performed consistently, since it is usually not the main focus of this experimental setup. Therefore, additive effects of possible hepatic toxicity and cutaneous side effects due to increased photon sensitivity as well as an increased risk of RT-related sequalae (such as radiation necrosis) need to be closely monitored in future and ongoing trials. Additionally, RDT should optimally be integrated into standard treatment, yet it remains unclear whether a combination with concurrent chemotherapy is feasible. Both ongoing clinical trials do not involve chemotherapy. Therefore, uncertainty remains regarding whether additional toxicity might derive from concurrent use of 5‑ALA in combination with hepatotoxic chemotherapy (e.g., temozolomide or CCNU in glioblastoma).

Lastly, the optimal RT dose and fractionation schedule for RDT remains unclear. In the context of modern radiation oncology, normofractioned RT remains common, but hypofractioned treatment with SRS or SBRT/SABR concepts is gaining increasing popularity, especially in palliative settings. The existing data range widely between single- and multi-fraction RT, with single fraction doses between 2 and 20 Gy, which correlate well with clinically implemented RT doses. Again, additional scientific effort is needed to sharpen the view on this detail, as current data do not allow for confident estimation of the superiority of one fractionation schedule over another. Notably, only one in vivo trial has replicated the course of standard RT in a rodent trial: Takahashi et al. used 60 Gy in 30 fractions for their GBM model and were able to demonstrate a radiosensitizing effect compared to normal treatment by the addition of 5‑ALA [70]. This pragmatic approach to investigate the addition of 5‑ALA to standard treatment might be pursued further for other tumor entities in the future, as this strategy delivers the most robust data regarding effectiveness and transferability to real-world practice.

While these limitations are varied and concern major details in the planning and conduction of ALA-RDT, the existing data deliver a promising foundation, and currently ongoing trials suggest a substantial chance to generate the necessary data regarding tolerability and effectiveness to further implement repeated applications of 5‑ALA as a radiosensitizer to increase the effectiveness of modern RT for different tumor entities.

## Conclusion

The capabilities of 5‑aminolevulinic acid as a radiosensitizer in radiodynamic therapy have been evaluated in a multitude of solid tumors. Preclinical data seem to deliver a solid basis for combined application of 5‑ALA and radiotherapy, as in vivo and in vitro models reveal the mechanism of enhancing radiation-induced cell damage, and tissue uptake is well documented based on experiences in PDT and fluorescence-guided resection. Uncertainties remain regarding the timing, dose, and combination of repeated ALA administrations in the context of modern radiation treatment concepts. Clinical trials and future scientific efforts will help to further explore the potential of this promising combination and are currently underway.

## Supplementary Information

Prisma checklist

## Data Availability

All data are publicly available via the PubMed database.
